# Cooperative Roles of SDF-1α and EGF Gradients on Tumor Cell Migration Revealed by a Robust 3D Microfluidic Model

**DOI:** 10.1371/journal.pone.0068422

**Published:** 2013-07-15

**Authors:** Beum Jun Kim, Pimkhuan Hannanta-anan, Michelle Chau, Yoon Soo Kim, Melody A. Swartz, Mingming Wu

**Affiliations:** 1 Department of Biological and Environmental Engineering, Cornell University, Ithaca, New York, United States of America; 2 Institute of Bioengineering and Swiss Institute for Experimental Cancer Research, School of Life Sciences, Ecole Polytechnique Fédérale de Lausanne (EPFL), Lausanne, Switzerland; University of Bergen, Norway

## Abstract

Chemokine-mediated directed tumor cell migration within a three dimensional (3D) matrix, or chemoinvasion, is an important early step in cancer metastasis. Despite its clinical importance, it is largely unknown how cytokine and growth factor gradients within the tumor microenvironment regulate chemoinvasion. We studied tumor cell chemoinvasion in well-defined and stable chemical gradients using a robust 3D microfluidic model. We used CXCL12 (also known as SDF-1α) and epidermal growth factor (EGF), two well-known extracellular signaling molecules that co-exist in the tumor microenvironment (e.g. lymph nodes or intravasation sites), and a malignant breast tumor cell line, MDA-MB-231, embedded in type I collagen. When subjected to SDF-1α gradients alone, MDA-MB-231 cells migrated up the gradient, and the measured chemosensitivity (defined as the average cell velocity along the direction of the gradient) followed the ligand – receptor (SDF-1α – CXCR4) binding kinetics. On the other hand, when subjected to EGF gradients alone, tumor cells increased their overall motility, but without statistically significant chemotactic (directed) migration, in contrast to previous reports using 2D chemotaxis assays. Interestingly, we found that the chemoinvasive behavior to SDF-1α gradients was abrogated or even reversed in the presence of uniform concentrations of EGF; however, the presence of SDF-1α and EGF together modulated tumor cell motility cooperatively. These findings demonstrate the capabilities of our microfluidic model in re-creating complex microenvironments for cells, and the importance of cooperative roles of multiple cytokine and growth factor gradients in regulating cell migration in 3D environments.

## Introduction

Tumor cell chemoinvasion within a 3D tissue, or chemoinvasion, is an important step in cancer metastasis [1], [2], [3]. Despite its clinical importance, the way tumor cells respond to chemical gradients within a complex microenvironment – particularly where multiple chemokines and growth factors coexist – is largely unknown [1], [2], [4]. Such gradients are the result of a highly complex and dynamic tumor microenvironment [5], [6] that consists of multiple cell types (e.g. stromal and immune cells), a heterogeneous extracellular matrix (ECM), and mechanical stress gradients that also drive interstitial flow [7]. Thus, to improve our understanding of how multiple exogenous factors affect tumor cell motility and chemoinvasion, robust *in vitro* models are needed that allow well-defined chemical gradients to be rapidly established and maintained across well-defined 3D cultures that are large enough to observe sufficient numbers of cells, with sufficient migration distances, to quantitatively evaluate the range of behaviors typically seen with tumor cell populations. Here, we asked how tumor cells respond to single vs. combined gradients of known chemoattractants using a newly developed 3D microfluidic culture model [8] with a more general goal of recreating a microenvironment that suppresses tumor cell dissemination.

The tumor microenvironment is spatially and temporally heterogeneous due to multiple chemokines and growth factors secreted by infiltrating leukocytes and surrounding stromal cells as well as by the tumor cells themselves [4], [9], [10]. Subsequently, extracellular signaling molecules form gradients that are critically regulated by infiltrating cells, interstitial fluid flow, and gradients in extracellular matrix density. Diffusion anisotropy and proteolytic degradation have been discussed in the current literature extensively [7], [11]. Amongst the chemoattractant signaling molecules that are known to be involved in tumor cell chemotaxis, CXCR4 (which binds stromal derived growth factor (SDF-1α or CXCL12) and EGFR (epidermal growth factor receptor) are notable in their relevance to the metastasis in many different cancer types, particularly breast cancer [4]. In Boyden chamber assays, human breast tumor cells have been shown to chemotact up gradients of both EGF [12], [13] and SDF-1α [14], [15]. Furthermore, EGFR signaling is well-known to enhance tumor cell motility [16], [17].

Still, researchers are only beginning to explore tumor cell invasion in more complex microenvironments [4], [18] such as those that exist not only in the primary tumor stroma but also niche sites for disseminated cells such as bone marrow or lymph nodes [6]. Furthermore, EGF-secreting macrophages were shown to be recruited to tumor-associated blood vessels that secrete SDF-1α from pericytes in a rat breast cancer model [19], [20]. Since such signaling pathways may have synergistic or antagonistic interactions, if any, it is important to build models and methods for qualitatively understanding cell response to complex environments, which is ultimately needed in future efforts aimed at building a predictive model for chemoinvasion in cancer [1].

Limitations of current models widely used to study chemotaxis or chemoinvasion, such as Boyden chambers, include (i) the lack of precise gradients that are stable in space or time [21], (ii) the lack of ability to differentiate chemotaxis from chemokinesis (i.e., enhancement of random motility but not directedness, which is less efficient for cell transport) [4], [11], and (iii) endpoint quality of the assay, which does not allow imaging during migration and thus misses information on the dynamics, distribution, and cell morphology during cell migration. Microfluidic chemoinvasion models have recently been introduced to overcome these limitations and create more physiologically relevant models [11], [22], [23], [24], [25], [26]. Additionally, current cancer cell chemotaxis studies using microfluidic models are largely limited to 2D, where cells are plated on a 2D substrate [27], [28]. 2D tumor cell chemotaxis is fundamentally different from that of 3D. In 2D, MDA-MB-231 cells use a mesenchymal migration strategy only because it requires integrin activities (or adhesion). In 3D, mammalian cells can either squeeze through the pores of the biomatrix via amoeboid motion or climb along the collagen fibers via mesenchymal motion. In the case of leukocytes in steady state conditions, cells have been found to move within collagen fibers via amoeboid motion and independent of integrin binding [29]. MDA-MB-231 cells have been shown to undergo mesenchymal-to-amoeboid transition when pericellular proteolysis is blocked [30].

In this study, we examine how tumor cell chemoinvasion behaviors can be affected by two competing chemical gradients, using a 3D microfluidic model with well-defined chemical gradients that are stable in space and time. A highly invasive and metastatic human breast cancer cell line, MDA-MB-231, was used because of the extent of characterization of this cell line [14], including its migration behavior in the presence of EGF or SDF-1α gradients using conventional Boyden chamber [12], [14], [31]. Additionally, the methodologies presented here are readily applicable to other tumor cells or to more complex tumor microenvironments.

## Materials and Methods

### Microfluidic Chemoinvasion Device Design and Characterization

**Figure 1:**
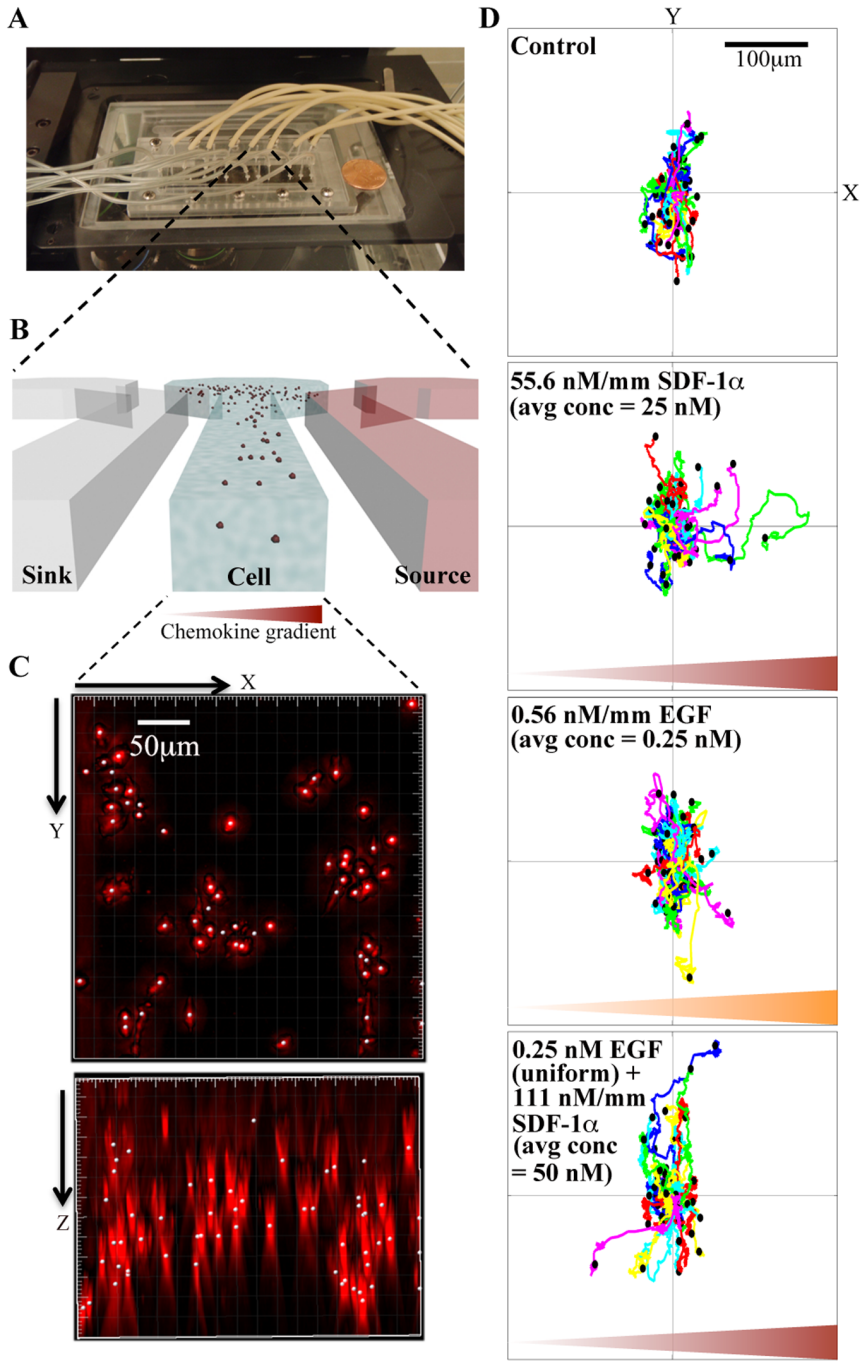
Microfluidic device setup and data acquisition. **A.** An image of the microfluidic device on the microscope stage. A penny is placed on the side for scale. **B.** Schematic illustration of the microfluidic device. Three parallel channels are patterned on a 1-mm thick agarose gel membrane. A stable linear gradient is generated across the center channel by flowing solutions of chemokine and buffer through the source and the sink channels respectively. A mixture of cells (1 million cells/ml) embedded in type I collagen (1.5 mg/ml) is seeded in the center channel. All three channels are 400 µm wide and 250 µm deep, and the ridges between the channels are 250 µm wide. **C.** 3D reconstruction of a z-stack of 65 images (5 µm each) of the cell-embedded collagen matrix viewed from x-y plane (top view) and the x-z plane (side view); scale bar, 50 µm. **D.** Cell trajectory plots (60 cells each) from the four conditions indicated. In the last panel, the uniform 0.25 nM EGF is generated by supplying 0.25 nM EGF solutions along the two side channels. Each colored line represents one cell trajectory tracked in 16 h.

A microfluidic chemoinvasion device previously developed in our lab was modified for this experiment [8], [32]. Chemoinvasion here is defined as tumor cell migration within 3D biomatrices under the influences of chemokines and growth factors. Briefly, four three – parallel – channel devices were patterned on a 1 mm thick agarose gel membrane using a silicon master. The agarose gel membrane was then placed on a 1 inch×1 inch glass slide and was sandwiched between a Plexiglas manifold and a stainless metal frame in a biohood. The operation principles are shown in the schematics in Figure 1B. Briefly, chemokine and buffer flow through two side channels respectively, and a linear chemokine gradient is established in the center channel via diffusion of chemokine molecules though the agarose ridges. The time for the gradient establishment depends on the diffusion coefficient of the molecules. For EGF (6.2 k*Da*) or SDF-1α (8.0 k*Da*), the molecular diffusion coefficient is about 111 µm^2^/s [33], it takes about 30 min to establish a steady gradient. To characterize the chemical gradients in the center channel of the device, we flow fluorescein-labeled dextran (0.1 mM, MW = 10 k*Da*, Invitrogen) and buffer through the two side channels, and then take the time-lapse fluorescence images of all three channels. The fluorescence intensity profile cross all three channels are used to represent the chemical concentration gradients (For more details please see ref. [8]).

### 3D Cell Culture

A malignant breast cancer cell line, MDA-MB-231, was obtained as a gift from the Cornell University Center on the Microenvironment and Metastasis. The basal medium for the cell line was DMEM (Invitrogen, Carlsbad, CA), supplemented with 10% FBS (Atlanta Biologicals, Lawrenceville, GA) and antibiotics (100 units penicillin and 100 µg streptomycin, Invitrogen). Cell cultures were maintained every 2–3 days at a T75 flask (Corning, Lowell, MA) with 5∼10% of initial confluency (percentage of cell area coverage) in a humidified, CO_2_-controlled incubator at 37**°**C. SDF-1α (10 µg/ml in PBS with 0.1% BSA) and EGF (200 µg/ml in 20 mM acetic acid) were purchased from R&D Systems (Minneapolis, MN) and stored at −20**°**C after reconstitution as instructed by the suppliers.

Type I collagen was extracted from rat tails (Pel-Freez, Rogers, AR) using a modified protocol [34] and stored at 5 mg/ml in 0.1% acetic acid at 4**°**C. Cell pellets from 50∼75% confluency from T75 cultures were re-suspended in DMEM with 10% FBS and then mixed at 1×10^6^ cells/ml with 1N NaOH (for pH∼7), 10X M199 and 0.15% collagen on ice. Cell numbers were counted using a hemocytometer (Bright-Line Hemocytometer, Hausser Sci., Horsham, PA). For a typical composition for 500 µl mixture, 150 µl 5 mg/ml collagen, 50 µl 10×M199, 3.3 µl 1N NaOH, and 296.7 µl cell culture at 1 million/mL cell concentration were mixed.

### Gel Filling and Device Setup

A volume of 20 µl of cell embedded collagen was introduced into the middle channel of each of the 4 devices using a gel-loading tip. All the inlets and outlets are plugged for preventing slow flow in the center channels during polymerization process. To polymerize the collagen gel, the device was placed in a 37**°**C incubator for at least 20 minutes of which the device was placed upside down for the first 7 minutes for better distributing cells in the z-direction. Cell distribution in 3D was visually confirmed using a bright field microscope (Nikon Eclipse TS100, Nikon Instruments, Melville, NY) right after the gelation (See Figure 1). Cells were incubated for 24 hours in the device so that cells will have time to attach to the matrix. We start imaging the cells at the same time when the chemical/buffer were introduced in the two side channels where we define t = 0. For a typical experiment, one device was used as a control where media were pumped through both side channels. Flows of three different chemical concentrations and buffers were introduced to the other three source and sink channels respectively. The flows ran at a rate of 1 µl/min through a medical grade tubing (ID = 0.51 mm, PharMed BPT, Cole-Parmer, Vernon Hills, IL) using a syringe pump (KDS230, KD Scientific, Holliston, MA) and a syringe (3 ml, BD, Franklin Lakes, NJ).

### Imaging and Data Analysis

For live cell imaging, the device was transferred onto an environmentally controlled microscope stage. The device was surrounded by a small Plexiglas chamber (120 mm×75 mm×45 mm) set at 37**°**C, 100% humidity and 5% CO_2_ (CO2-200, In Vivo Scientific, World Precision Instruments, Inc., Sarasota, FL). The microscope stage were surrounded by a temperature controlled chamber (Weather Station, Precision Control LLC) set at 37**°**C. For each experiment, we typically imaged 8 positions (2 on each device, with 4 devices on one chip) using the x-y controlled stage (OptiScan II, Prior Scientific, Inc., Rockland, MA). The images were captured every 5 minutes for 16 hours using the bright field microscopy (20×objective, Olympus IX81, Center Valley, PA), an image acquisition software SlideBook (Intelligent Imaging Innovations, Inc., Denver, CO) and a CCD camera (Orca-ER, Hamamatsu, Bridgewater, NJ). For the data reported here, we imaged one plane close to the center of the channel in the vertical direction. These experiments were repeated at least once.

**Figure 2:**
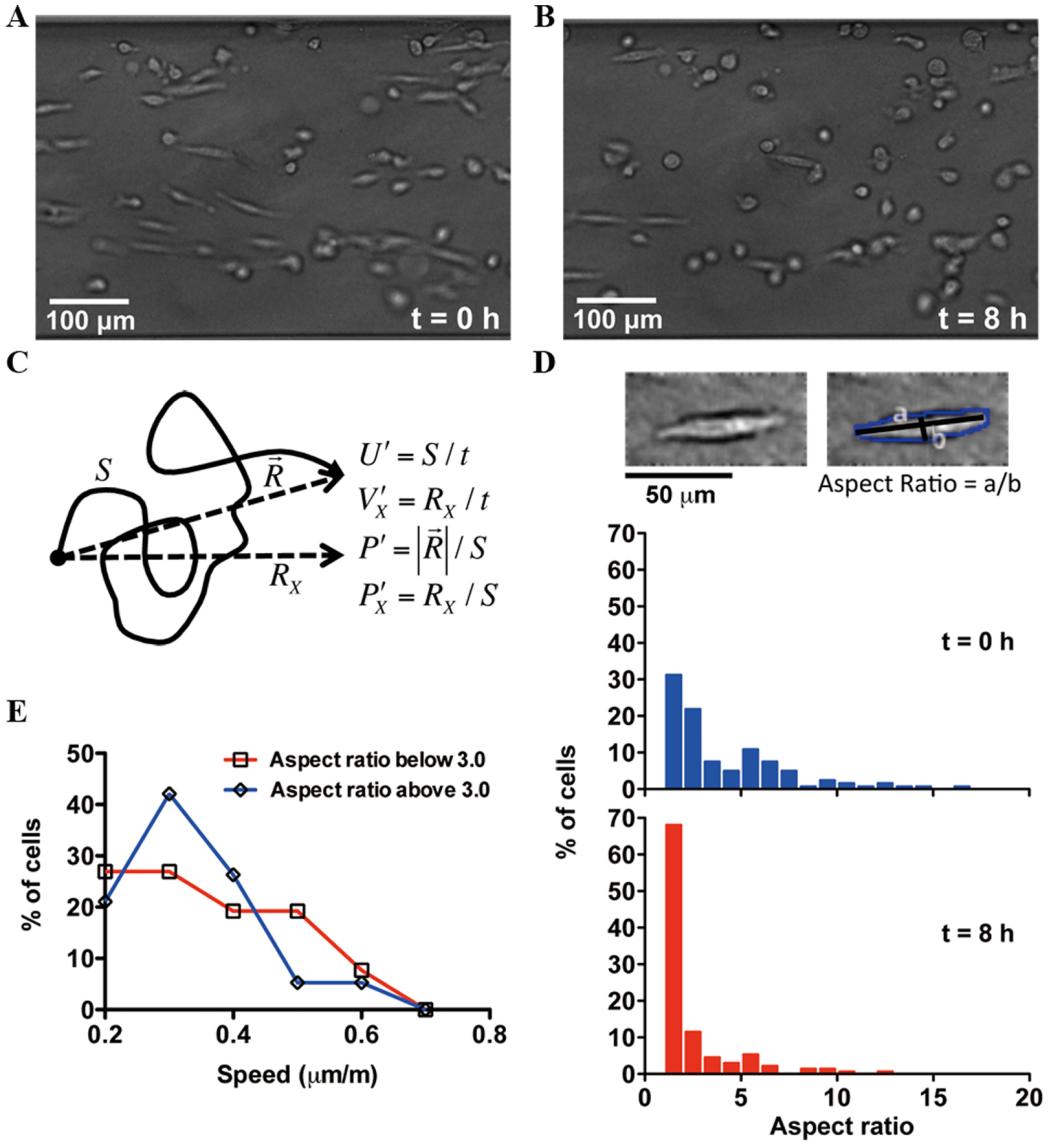
Plasticity and heterogeneity of tumor cell morphology and motility behavior. **A–B**. Bright field images of MDA-MB-231 cells embedded in 3D collagen matrices within the microfluidic channel at t = 0 and 8 h. Here, t = 0 is defined as the time when buffer and 100 nM SDF-1α solution were introduced into the two side channels. Cells were pre-incubated for 24 hours after seeding before the introduction of the gradients. **C.** Graphical description of cell speed 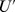, cell velocity along the gradient direction 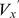, persistence length 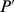, and gradient-directed persistence length 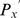. **D.** Graphical description of aspect ratio. Distribution of cell aspect ratios at t = 0 and 8 h. **E.** Distribution of cell speed of elongated cells (aspect ratio larger than 3) and amoeboid-like cells (aspect ratio less than 3).

Cell trajectories were obtained first using a Manual Tracker in ImageJ (National Institutes of Health) from the time series images as shown in Figure 1D. Cell speed 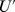 (length of the trajectories divided by time), the velocity 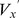 (displacement along the gradient direction divided by time), cell persistence length 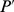 (the displacement of a cell trajectory divided by the length of the trajectory), and the cell persistence length along gradient direction 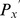 (the displacement of a cell trajectory along the gradient direction divided by the length of the trajectory) were then computed from the cell trajectories of 16 hour duration (See Figure 2E) using an in house Matlab program (The MathWorks, Inc., Natick, MA). Here, only motile cells (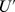 >0.2 µm/min) were included for further data analysis, which usually accounted for ∼50% of the total cell population. The average speed, velocity and persistence length were computed from about 120 or more cells under the same chemical gradient, which usually come from at least two separate experiments. To minimize the experiment-to-experiment variation, speed 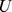 is normalized by the average speed of the control cells (no chemical gradients), and velocity along the direction of gradient, 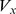, is computed as the average velocity along x-direction subtracted by that of the control group, and divided by the average speed of the control. Persistence length *_P_* is normalized by the average persistence length of the control group. Persistence length along the gradient direction 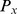 is normalized by subtracting that of the control group. Nonparametric *t*-test compared to the control group (Mann-Whitney test) within Prism (GraphPad Software, Inc., La Jolla, CA) was used for statistical analysis.

## Results and Discussion

### Microfluidic Device Setup, 3D Cell Culture and Dynamic Cell Tracking

Chemical gradients were generated using a recently developed microfluidic device (Fig. 1A–B) [8], [24]. Briefly, three parallel channels were patterned on a 1 mm thin agarose gel membrane. Medium containing either SDF-1α, EGF, or neither was introduced to each of two side channels, and allowed to form gradients by diffusion across the agarose gel ridges between the channels. Cell-seeded type I collagen was introduced into the center channel. This device has been recently characterized for its ability to generate stable and well-defined gradients both computationally and experimentally using FITC conjugated proteins [8], [24], which was used to explore dendritic cell chemotaxis previously [8], [24]. Various chemical gradients were generated in the center channel by changing the chemical concentrations in the source and sink channels. The chemical gradient is calculated as the difference of the chemical concentrations in sink and source channels divided by the distance between the source and sink channels (i.e. 900 µm). The average chemical concentration is computed as the average of the chemical concentrations in sink and the source channels. A uniform concentration in the center channel is generated by supplying the same chemical concentration solution in both side channels.

The 3D cell culture consisted of 10^6^ tumor cells/ml embedded in type I collagen extracted from rat tail [34]. Different concentrations (0.15, 0.25 and 0.35%) of collagen were compared, and we measured the average cell speed to be 0.42±0.02, 0.30±0.02, and 0.26±0.02 µm/min, respectively. We therefore used 1.5 mg/ml collagen for all the experiments shown here. In our culture model, cells remained evenly distributed after collagen polymerization (Fig. 1C) in part aided by inverting the device upside-down during the first 7 min of collagen polymerization [8].

*In vitro* collagen matrices are substantially different than those *in vivo*, specifically being more simple in composition, less dense in collagen, and more spatially homogeneous. A high collagen density (similar to that of breast tumor *in vivo*) cannot be used for *in vitro* assays, since tumor cell migration is suppressed by the dense and spatially homogenous biomatrix network. However, it has been shown that the *in vivo* tumor microenvironment is highly heterogeneic in terms of spatial distribution of the collagen fibers. Multiphoton imaging of breast tumor cell migration in mouse model shows that fast and persistent tumor cell migration is often associated with three factors – the lack of dense collagen network, amoeboid motion, and the contact of cells with ECM fibers [35], [36]. It is thus important to place tumor cells in the context of a collagen matrix for *in vitro* studies, and processes like tumor cell chemoinvasion can be mimicked with close proximity to the *in vivo* situation in such matrices [24], [29], [37].

Cell movements were characterized in real time and space by taking a time sequence of images of migrating cells, and subsequently tracking the cell positions at various time points. Examples of cell tracking process are shown in Movie S1 and Movie S2. Figure 1D shows cell trajectories of 16 h duration under four different chemical gradient conditions. These polar plots were formed by placing the first cell position of each cell track at the center coordinate. It should be noted that although the experiments were conducted in 3D, the cell tracking was carried out using images taken at a fixed 2D (or x–y) plane. Figure 1D shows that tumor cells migrated randomly under control conditions (no chemical gradients), were chemoinvasive towards SDF-1α gradients, and displayed chemokinesis (or enhanced motility) in the presence of EGF gradients as well as in the presence of both EGF and SDF-1α gradients.

### Plasticity of Tumor Cell Morphology and Migration Behavior under Changing Microenvironments

The morphology and migration of the MDA-MB-231 cells were followed when flows were introduced to the two side channels. When MDA-MB-231 cells were initially cultured in type I collagen matrix for 24 hours with no flow along the two side channels, the majority of motile cells exhibited a typical elongated (or mesenchymal-like) morphology, in contrast to a more rounded (or amoeboid-like) morphology (See Figure 2A,B). Initially, about 50% of motile cells had aspect ratios less than 3. After 8 hours flowing buffer or chemokine through the two side channels, the majority of motile cells (∼80%) had aspect ratios less than 3 (See Figure 2B, D). Figure 2D shows that the aspect ratio of the cells decreased with time. Note that we exclude the potential stress under the microscope based on the observations that cells are motile as shown in Movie S1 and Movie S2. In addition, we found that i) the cells without flow in the side channels maintained a high aspect ratio in the microscope stage; and ii) the majority of the cells with flow in the side channels for the initial culture period (24 h) in a conventional incubator showed a similar decrease in the aspect ratio.

### Tumor Cell Chemoinvasion in SDF-1α Gradients Follows the Ligand – Receptor Binding Kinetics

**Figure 3:**
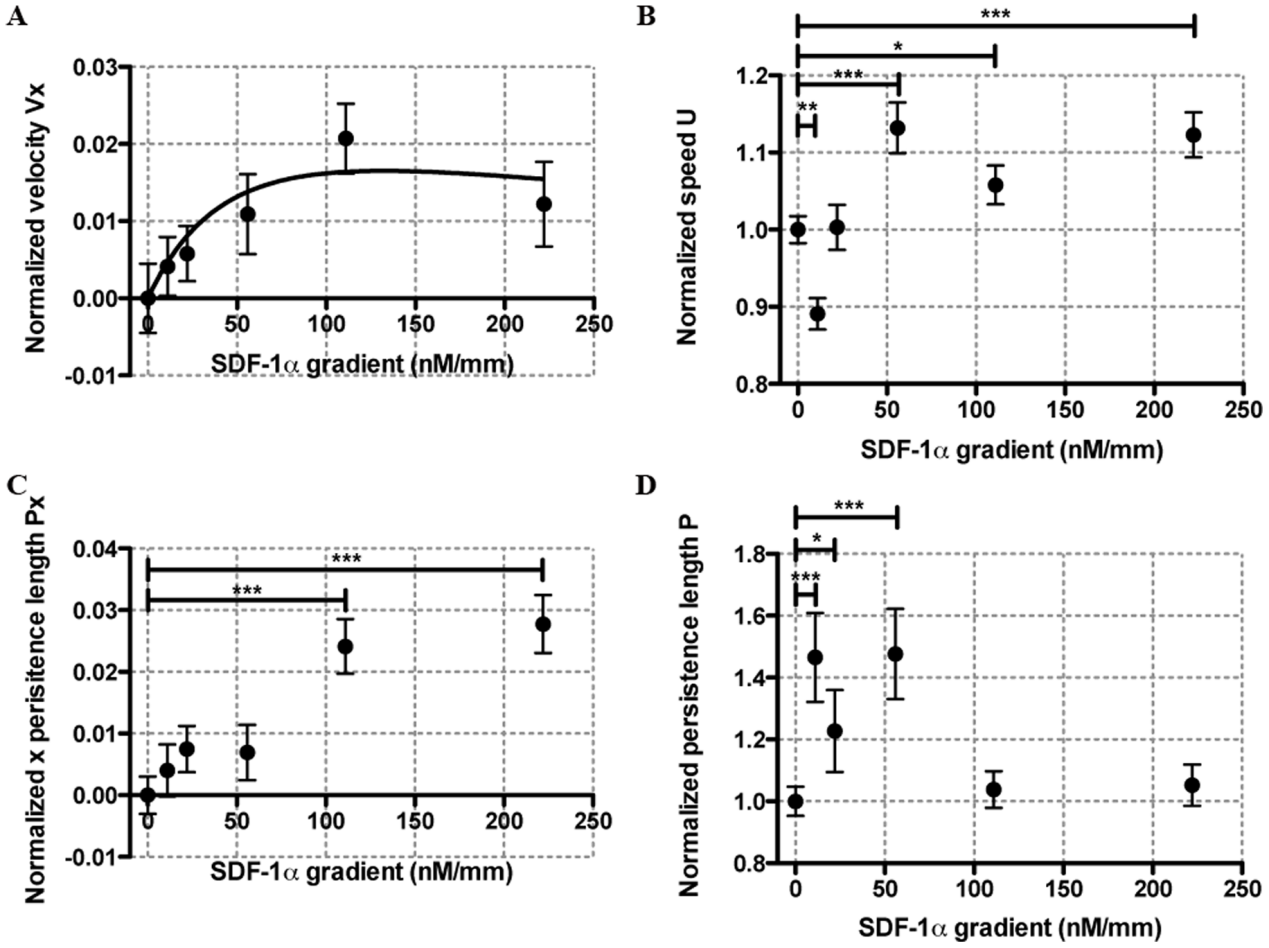
Chemoinvasive and chemokinetic behavior of tumor cells to linear SDF-1α gradients. **A.** Average cell velocity 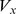 along the SDF-1α gradient as a function of the SDF-1α gradient 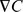. Solid line is a fit to the ligand – receptor binding kinetics 
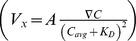
. **B.** Average cell speed as a function of the SDF-1α concentration gradient. **C.** Average persistence length along the gradient direction 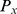 as a function of SDF-1α concentration gradient. **D.** Average persistence length 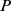 as a function of SDF-1α concentration gradient. The stars were obtained using a nonparametric *t*-test compared to the control group (Mann-Whitney test with * for 0.01<*p*<0.05, ** for 0.001<*p*<0.01, and *** for *p*<0.001).

Figure 3A shows the average cell velocity along the gradient 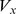 as a function of SDF-1α gradient. Note that 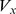 peaks at SDF-1α gradient of 111 nM/mm. Early work from our groups [24] and others [38] have shown that immune cell chemoinvasion follows a ligand – receptor kinetics, indicating that cell chemoinvasion is governed by the difference of the ligand – receptor bound states at the front and rear of the cell. Therefore, we fitted the 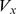 versus SDF-1α gradient data to the ligand – receptor association kinetic equation, more specifically the difference of the ligand – receptor bound states at the front and rear of the cell, 
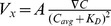
, where 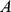 is a constant and 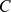 is the SDF-1α concentration (See Figure 3A). The fitted data provides a ligand – receptor association constant 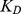 = (59.2±38.3) nM. This agrees well with the reported literature value of 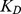 = (55±15) nM, where the kinetic association constant of SDF-1α and CXCR4 was measured using an elegant fluorescence resonance energy transfer (FRET) method [39], [40].

### Tumor Cells Display Mild Chemokinesis in SDF-1α Gradients

We first looked at the fraction of migrating cells (defined as cell speed >0.2 µm/min), and observed no visible changes, 0.64±0.05 for control vs. 0.62±0.04 for 111 nM/mm SDF-1α. We then plotted the average cell speed under various SDF-1α gradients. Figure 3B shows that cells have no significant speed increase when SDF-1α gradient was less than 56 nM/mm (or an average SDF-1α concentration of 25 nM) and an increase of speed, about 7–13%, when SDF-1α gradient was equal or greater than 56 nM/mm. Measurements of persistence lengths along×direction also demonstrate the chemotactic behavior of tumor cells along the SDF-1α gradients (Figure 3C). It is interesting to note that the persistence length was significantly higher (20–40%) at low SDF-1α gradients (less or equal to 56 nM/mm) (Figure 3D).

### Tumor Cells Showed no Significant Chemoinvasion, but Mild Chemokinesis in EGF Gradients

**Figure 4:**
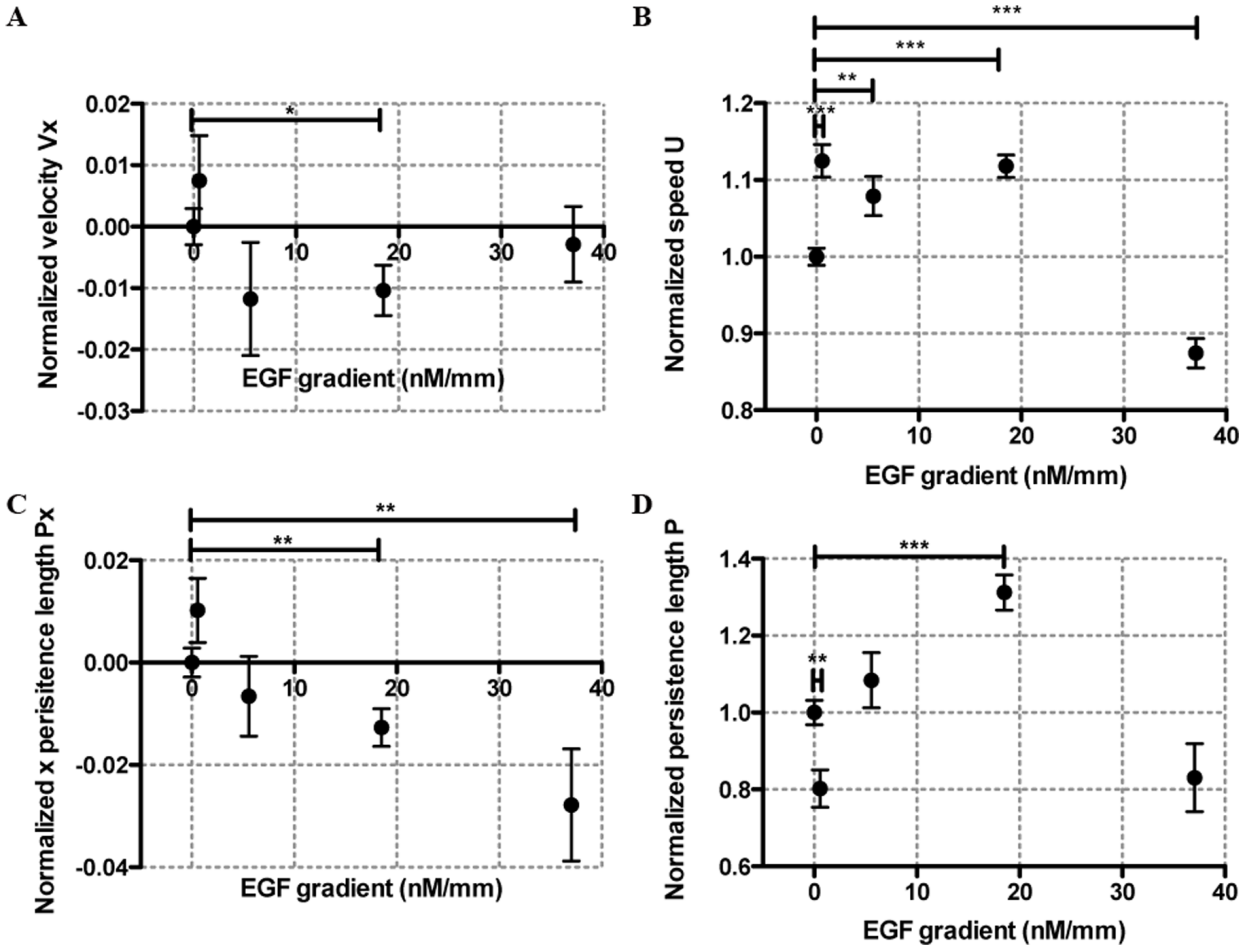
Tumor cells display no chemoinvasion but mild chemokinesis in linear EGF gradients. Average cell velocity 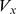 along the EGF gradient (**A**), average cell speed 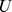 (**B**), average persistence length along the EGF concentration gradient 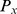 (**C**) and average persistence length 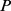 (**D**) as a function of EGF gradients. The stars were obtained using a nonparametric *t*-test compared to the control group (Mann-Whitney test with * for 0.01<*p*<0.05, ** for 0.001<*p*<0.01, and *** for *p*<0.001).

The 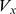 versus EGF gradients plot in Figure 4A shows that no statistically significant chemoinvasion behavior were observed under four different EGF gradients; in contrast to previous report that EGF is a chemo-attractant for human breast tumor cells (MDA-MB-231) using Boyden chamber assays [12], [41], Dunn Chamber (a 2D assay where cells are plated on a substrate) [42] and rat breast tumor cells [43]. Cell average speed has an increase of about 8–12% for EGF gradient of 0.56, 5.56 and 18.52 nM/mm or average EGF concentration of 0.25, 2.5 and 8.33 nM. This is consistent with previous report that a small fraction (2–5%) of the EGF receptors display a high EGF-binding affinity (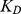 = 10–100 pM), whereas the majority of the receptors (95–98%) display a lowered association constant (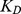 = 2–5 nM) obtained by a ^125^I labeled EGF binding assay [44], [45]. It should also be noted that EGFR is known to internalize in the presence of ligand binding, which may also contribute to the behavior observed in Figure 4B [46].

The difference of our chemoinvasion results in EGF gradients to those reported in the literature using Boyden chamber is likely caused by the fact that (i) Boyden chambers do not distinguish chemotaxis versus chemokinesis [12], [41]; (ii) 2D chemotaxis is fundamentally different from 3D chemoinvasion [28], [42]. In 2D, cells exhibit large focal adhesion complexes, and their migration behavior depend critically on the integrin binding sites. In 3D, motile MDA-MB-231 cells displayed mostly amoeboid-like (or rounded) cell morphology (See Figure 2), and they migrated by squeezing through the collagen fiber pores. For leukocytes in steady-state conditions, amoeboid cell migration within a 3D environment has been found to be integrin-independent [29]. It has also been reported that the organization of focal adhesion proteins may be different in 2D vs. 3D conditions [47]. Further studies controlling integrin expression will be needed to elucidate the differential roles of integrin in 2D versus 3D chemoinvasion.

Using a 2D microfluidic model, it was reported that EGF gradient steepness played a critical role in MDA-MB-231 cell chemotaxis [28]. Although it is difficult to compare the results from a 3D microfluidic model here directly with those of a 2D model, we do not exclude the possibility that a steeper EGF gradient may stimulate a chemotactic response. The gradient shapes could be critical in the case of aggregating receptor systems such as EGFR, suggesting that the difference in fractional receptor activation is more important than the difference in fractional receptor occupancy [48].

The persistence length along the gradient direction 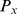 was decreased in the presence of EGF gradients (Figure 4C), while the persistence length 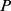 under various EGF gradients (Figure 4D) did not display a general pattern, it decreased at an EGF gradient of 0.56 nM/mm (or average concentration of 0.25 nM), and increased at EGF gradient of 18.2 nM/mm (or average concentration of 8.33 nM).

### Uniform Background of EGF Abrogates Chemoinvasion of MDA-MB-231 Cells in SDF-1α Gradients; EGF and SDF-1α Cooperatively Modulate MDA-MB-231 Cell Motility

**Figure 5:**
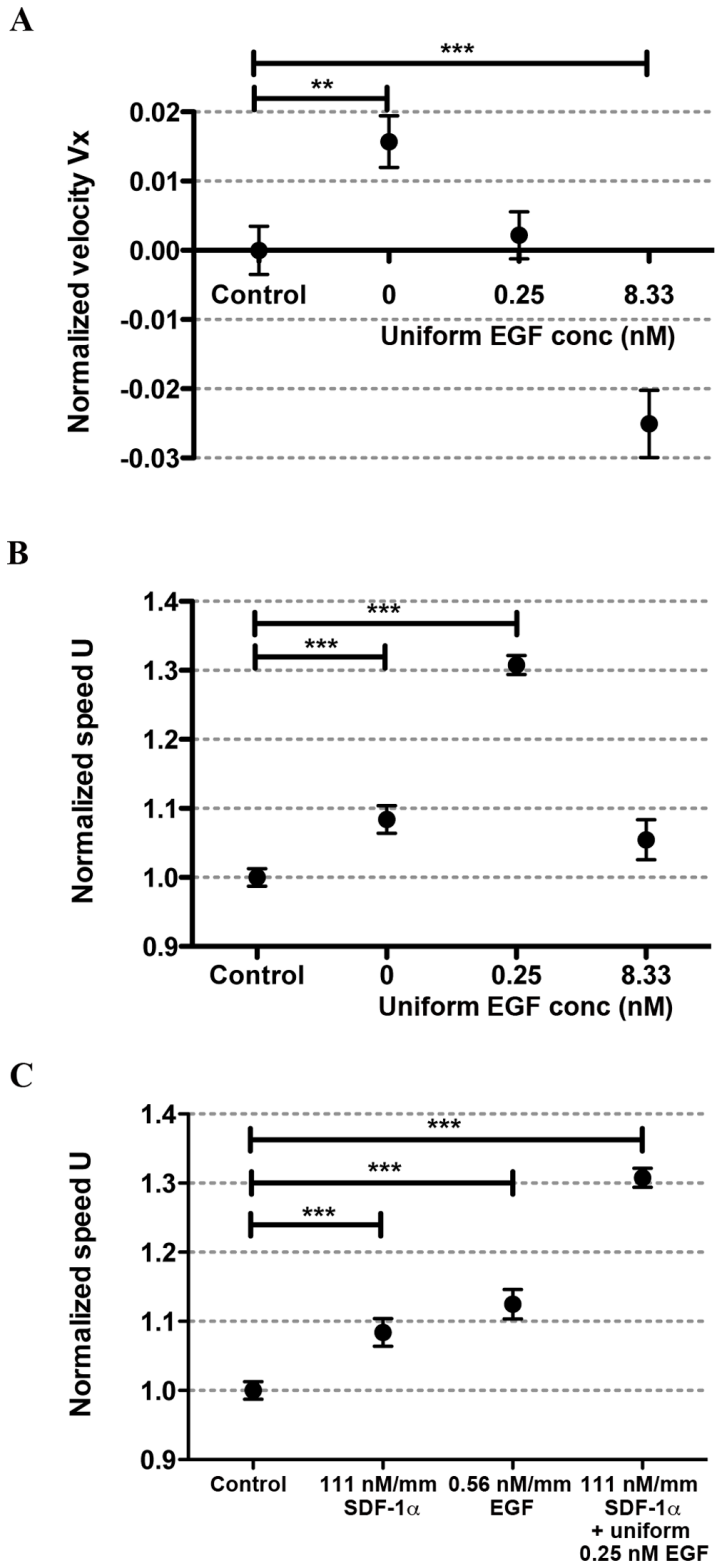
Cooperative roles of EGF and SDF-1α in tumor cell chemoinvasion. Average cell velocity 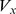 (**A**) and speed 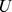 (**B**) in the presence of a SDF-1α gradient of 111 nM/mm and a uniform EGF concentration of 0, 0.25 or 8.33 nM. Control conditions were without SDF-1α and EGF. **C.** Average cell speed under indicated conditions. The stars were obtained using a nonparametric *t*-test compared to the control group (Mann-Whitney test with * for 0.01<*p*<0.05, ** for 0.001<*p*<0.01, and *** for *p*<0.001).

Surprisingly, we found that tumor cell chemoinvasion up a gradient of SDF-1α (111 nM/mm) was abrogated by the presence of a uniform background of 0.25 nM EGF (Fig. 5A). Furthermore, when EGF concentration was increased to 8.33 nM, tumor cells actually displayed chemorepulsive behavior to the SDF-1α gradient (Fig. 5A).

Previous work using a 2D microfluidic model demonstrated that exogenous EGF is required for facilitating MDA-MB-231 cell chemotaxis in SDF-1α gradients [27]. We argue that the difference of our observation and their work comes from three factors. (i) 2D cell migration is fundamentally different from 3D cell migration as stated above; (ii) Autocrine signals are washed away in the flow based 2D device, while they retained in the diffusion based 3D device. It has been demonstrated that autocrine EGF influences the persistence of epithelial cell migration [49]; (iii) Flow based 2D device provides steeper and nonlinear gradient profile, while the diffusion based 3D device provides linear gradient profile.

Figure 5B shows that EGF and SDF-1α cooperatively modulate cell motility. Here we show the average cell speed under the same chemical gradient condition as those shown in Figure 5A. With EGF (0.25 nM) alone, the cell speed increased ∼9%; with SDF-1α gradient (111 nM/mm) and EGF background (0.25 nM average concentration), the cell speed increased ∼30% in comparison to the control group. Further increasing EGF concentration to 8.33 nM inhibited this phenomenon.

Figure 5C further demonstrate that EGF and SDF-1α cooperatively modulates tumor cell motility. Here, a ∼9% speed increase is observed when the cells are in the presence of SDF-1α only, and 11% increase when the cells are in the presence of EGF only, but a ∼30% increase when both SDF-1α and EGF are present. It should be noted that this motility enhancement is abrogated at high EGF concentration (8.3 nM) when all the EGFR receptors are saturated.

Cross signaling between CXCR4 and EGFR has been found to stimulate cancer cell growth previously [50], however its impact on cancer cell migration in 3D microenvironment has not been explored [18]. Results presented here demonstrate the capability of a 3D microfluidic *in vitro* model in presenting complex chemical gradients to cancer cells, and the importance of the cross signaling between two important receptors CXCR4 and EGFR on tumor cell dissemination.

In summary, we present experimental work on how breast tumor cells (MDA-MB-231) were regulated by single or dual gradients in 3D environment to drive directed invasion, which was previously unknown. We demonstrated that tumor cell chemoinvasion in SDF-1α (ligand to CXCR4) gradients follows a general ligand – receptor binding dynamics, highlighting the importance of the ligand – receptor association constant 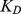. Not only EGF gradients alone do not cause chemoinvasion, the presence of EGF background abrogate the chemoinvasive behavior of tumor cells in SDF-1α gradients; in contrast to the observations in a 2D environment [18], [42]. Cooperatively, EGF and SDF-1α modulates tumor cell motility. This work highlights the importance of studying tumor cell chemoinvasion within a physiologically realistic, 3D, microenvironment, and provides a general framework for future data driven theoretical modeling of the 3D tumor cell chemoinvasion processes within a complex microenvironment.

## Supporting Information

Movie S1 **Tracking MDA-MB-231 cells for 16 hours in control.** Tumor cell morphology heterogeneity is shown in the movie. No directed cell migration is observed in the control movie. The channel width is 400 µm and the time between two consecutive images is 8 minutes.
(AVI)

Movie S2 **Tracking MDA-MB-231 cells for 16 hours under a 56 nM/mm SDF-1α gradient.** Tumor cell morphology heterogeneity is shown in the movie. Chemotactic motion towards the high concentration of SDF-1α (right-side) side is observed. The channel width is 400 µm and the time between two consecutive images is 8 minutes.
(AVI)
